# Epileptiform discharges in the anterior thalamus of epilepsy patients

**DOI:** 10.1016/j.isci.2024.109582

**Published:** 2024-03-27

**Authors:** Zsófia Jordán, Johanna-Petra Szabó, Anna Sákovics, Anna Kelemen, László Halász, Loránd Erőss, Dániel Fabó

**Affiliations:** 1 Epilepsy Unit, Department of Neurosurgery and Neurointervention, Faculty of Medicine, Semmelweis University, 1145 Budapest, Hungary; 2 Member of the ERN EpiCARE, Budapest, Hungary; 3 János Szentágothai Neuroscience Program, School of PhD Studies, Semmelweis University, 1085 Budapest, Hungary; 4 Lendület Laboratory of Systems Neuroscience, Institute of Experimental Medicine, 1083 Budapest, Hungary; 5 András Pető Faculty, Semmelweis University, 1125 Budapest, Hungary; 6 Functional Neurosurgery Unit, Department of Neurosurgery and Neurointervention, Faculty of Medicine, Semmelweis University, 1145 Budapest, Hungary; 7 Department of Neurology, University of Szeged, 6720 Szeged, Hungary

**Keywords:** Health sciences, Clinical finding, Medical tests

## Abstract

Anterior thalamus (ANT) deep-brain stimulation (DBS) is an approved therapy for drug resistant epilepsy. We aimed to identify interictal epileptiform discharges (IED) in the ANT and to investigate their relationship with surface IEDs. Fifteen patients were monitored for two consecutive nights with externalized thalamic leads to analyze the intrathalamic epileptiform activities (TIED). Forty-six % of all contacts were located within the ANT. We found that all the responders had TIEDs within the ANT, while this held true only for 44% of the non-responders. The overall response rate (RR) at 1-year follow-up was 40%, while it was 44% in bilateral ANT hit patients and 45% in epileptic focus side hit. However, in case of TIEDs present in the focus side the RR reached as high as 71%. TIED activity may prove the pathophysiological connection to the seizure focus, and stimulation of this area might have a better suppressing effect on seizures.

## Highlights

• Ventral part of ANT performed TIED activity in 92%
• 100% of responders vs. 44% of the non-responders had spike type TIEDs within the ANT
• Response rate on the focus side was 40% vs. 71%, without or with TIEDs, respectively
• TIED activity in ANT may prove the pathophysiological connection to the seizure focus

## Graphical abstract

**Figure:**
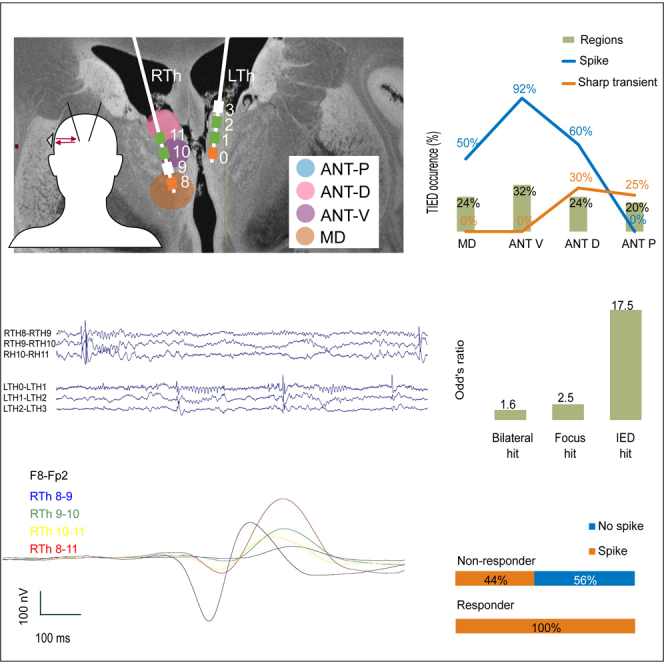

## Introduction

Anterior thalamus (ANT) deep-brain stimulation (DBS) is an approved neuromodulation therapy for drug resistant epilepsy.^1^ The efficacy is based on preclinical,^2^^,^^3^ open label,^4^^,^^5^ and double-blind clinical studies^6^ showing that the lesioning or high frequency stimulation of the ANT can reduce or alleviate epileptic seizures and long lasting neuromodulatory action was postulated in the limbic circuit.^7^^,^^8^ Beside ANT other thalamic areas, like centromedial, dorsomedial, and pulvinar nuclei were investigated as potential DBS targets with various results, but none of them were approved yet.^9^ For review see Martín-López D et al.^10^

Although the pathological role of the thalamus in both focal and generalized epilepsies with spike and wave discharges (SWD) was hypothesized and proven as early as the mid 1940’s,^11^ the mechanism of action of ANT-DBS is not explained yet. Although ANT is a prominent hub in the Papez circuit^12^^,^^13^ and refractory temporal lobe epilepsy is considered as positive selection criterium for ANT-DBS,^14^ no studies reinforced the idea that only epilepsies affecting limbic areas are responsive to the therapy.^15^ The overall response rate varies around 72% (46–90%)^15^ that tends to improve over years of chronic stimulation, regardless of the location of seizure focus.^15^^,^^16^^,^^17^^,^^18^

The ANT is located bilaterally near the anterior aspect of the third ventricle close to the foramen Monroi just beneath the lateral ventricle. The four contact DBS leads (Medtronic 3387 and 3389 leads, Medtronic Corp, MN, USA) are introduced via stereotactic methods. The most common route to approach the target goes transventricular from a superior frontal entry point. In cases where transventricular approach is not possible, it can be omitted using an inferior frontal entry point. Via these routes, 1, 2, or 3 contacts can be introduced into the ANT.^19^^,^^20^ There is another dorsal trajectory with a parietal entry point. Due to the antero-posterior orientation of the nucleus, this approach has the potential to place all four contacts into the ANT.^19^ There is some evidence that intraoperative unit recording can aid targeting of the ANT during surgery but seems not to be related to clinical outcome.^21^^,^^22^

The final position of the stimulated contacts is essential to obtain the best efficacy. According to former studies the best positions were in the anteroventral ANT in close vicinity to the mammillothalamic tract.^23^^,^^24^

Other studies targeting pre- or intraoperative factors supporting patient selection or further refinement of the lead position have not yielded useful biomarkers to predict good therapeutic response yet. One study measuring diffusion tensor imaging-based brain connectivity between the stimulated volume and the rest of the brain demonstrated that higher connectivity with the areas of the resting state network coincided with better response.^25^ This result provided further evidence that ANT maintains functional connections to extra limbic cortical areas too that have outstanding importance in influencing the epileptic focus. It has also been shown that low- and high frequency stimulation of the ANT decreases cortical excitability. At the same time, it provides information on cortical areas engaged by stimulation and supports the optimization of stimulation parameters,^26^ which might contribute to better clinical outcome.^27^

Our group performed an analysis on a patient cohort overlapping with the present report and found that decreased ongoing high frequency oscillation (HFO) content measured in the ANT versus the surrounding thalamic structures coincided with better therapy outcome.^28^ Interictal epileptiform discharges (IED) together with HFO are hallmark EEG phenomena characteristic for the epileptogenic transformation of the cortex,^29^ but the existence of IEDs in subcortical locations was long debated. Recent studies demonstrated the presence of IEDs and seizures on local field potential (LFP) recordings from the ANT.^30^^,^^31^ Thalamic IEDs (TIED) were identified in all the temporal lobe cases and the TIED frequency correlated with therapy outcome. In addition, newly emerging machine learning approaches were able to differentiate cortically generated seizures from interictal states based on ANT spectral changes.^32^

In this study, we aimed to assess the validity of LFP recordings of TIEDs within the ANT to predict DBS response.

## Results

### Clinical characteristics: Predominance of temporal lobe cases

**Figure 1:**
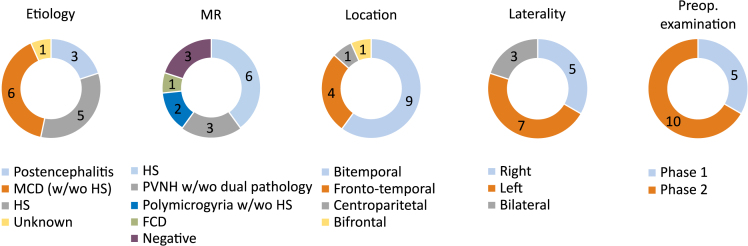
Characteristics of clinical data Proportions regarding etiology, MR findings, location, laterality dominance and preoperative examination of investigated epilepsy patients.

The clinical characteristics of the fifteen involved patients are presented in Figure 1. The locations of epileptogenic foci were: bitemporal 60% (*n* = 9), fronto-temporal 26,7% (*n* = 4), centroparietal 6,65% (*n* = 1), and bifrontal 6,65% (*n* = 1). The laterality was right or right dominant 33% (*n* = 5), left or left dominant 47% (*n* = 7), or without laterality dominance 20% (*n* = 3). The etiology included postencephalitis 20% (*n* = 3), hippocampal sclerosis (HS) 33% (*n* = 5), malformation of cortical development with or without HS 40% (*n* = 6), and unknown 7% (*n* = 1). The MRI finding included HS 40% (*n* = 6), periventricular nodular heterotopia with 13% (*n* = 1) or without dual pathology 7% (*n* = 2), polymicrogyria 7% with (*n* = 1) or without HS 7% (*n* = 1), focal cortical dysplasia 7% (*n* = 1), and no alteration 20% (*n* = 3).

**Figure 2:**
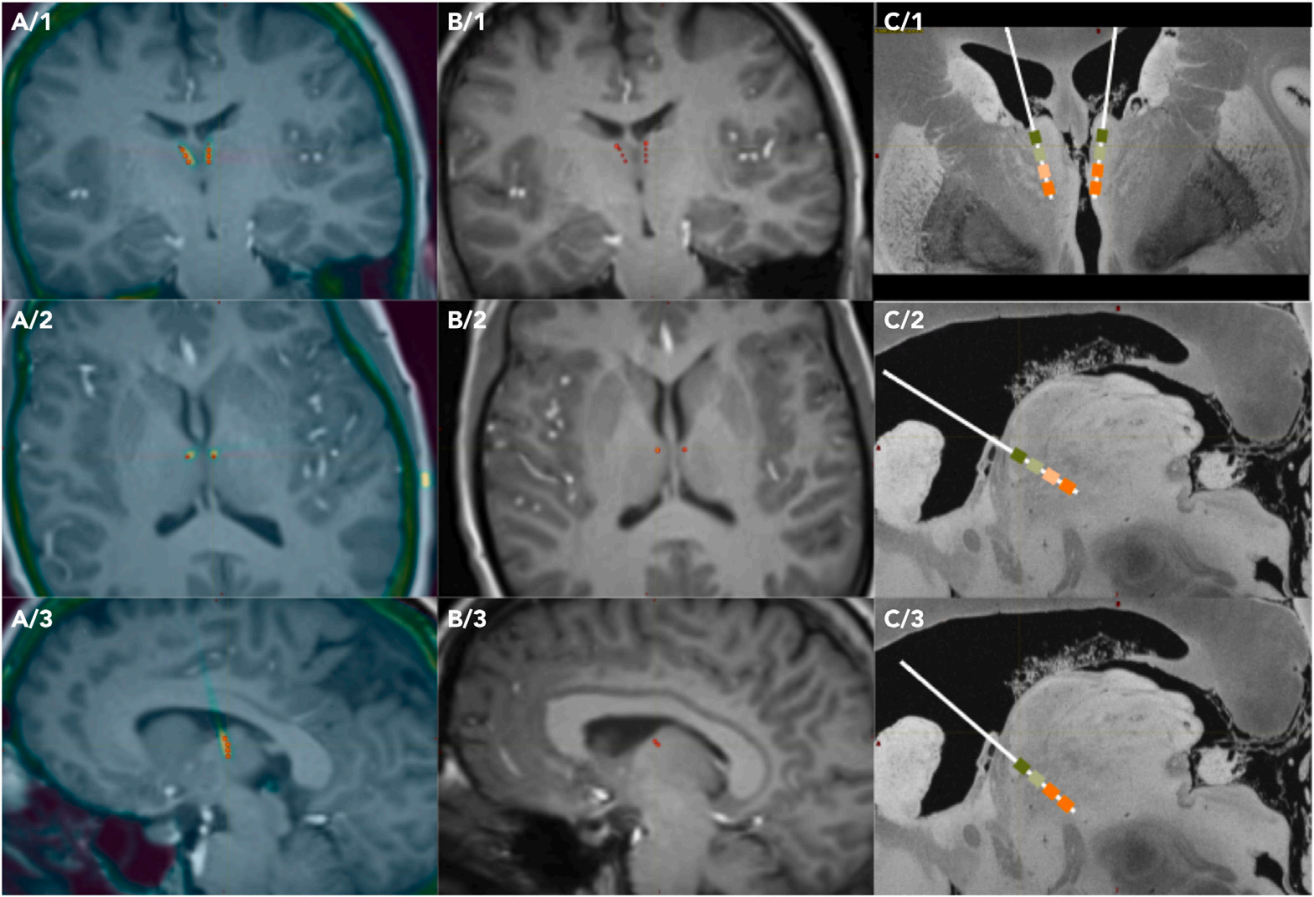
Reconstruction of DBS lead positions within the anterior thalamus (ANT) Anatomical localization of contacts in case of an example patient (Pt9), and position of DBS leads. (A1–A3) images show preoperative MRI (grayscale) and postoperative CT (color coded) fusion, with automatic contact localization results (red dots) in different section planes coronal (A1), axial (A2), and sagittal left (A3). (B1–B3) MRI-contact fusion images in the same planes as in column A. Only preoperative MRI and contact location data are selected for further visual analysis. (C1–C3) result of the visual analysis. A 7T MRI of a control case (downloaded from common database^39^) was used for demonstration purposes, and a size adjusted lead model was placed visually based on a group consensus (ZsJ, LH, LE, DF) of the contact locations seen on the MRI-contact fusion images. Selected planes are coronal (C1), sagittal right ANT (C2), and sagittal left ANT (C3). Green rectangles mark electrodes within the ANT, orange rectangles mark the ones in the mediodorsal thalamus (MD).

Figure 2 shows an example of the reconstruction of DBS lead positions within the anterior thalamus (detailed in STAR Methods). Based on this electrode contact reconstruction nine patients succeeded to have contacts bilaterally within the ANT and the remaining six had only unilateral ANT hits, resulting that 80% of the implanted leads (*n* = 24) was a hit and 20% (*n* = 6) missed the target letting the lead remaining intraventricular. The 24 intranuclear leads resulted in 55 contacts (46% of all) finally located within the ANT. There were no patients with bilaterally missed ANT. The intranuclear leads were implanted from different trajectories: frontal transventricular in 54% (*n* = 13), parietal transventricular in 38% (*n* = 9), and lateral in 8% (*n* = 2). The different trajectories’ ANT hit ratio was the following: frontal 52%, parietal 69%, and lateral 38%. The mediodorsal nucleus of the thalamus (MD) was hit with 21 contacts (18%) in 10 patients. After parcellation of the ANT into ANT-V, ANT-D, and ANT-P parts, these 76 intrathalamic contacts (ANT and MD together) were identified being placed in 41 thalamic regions altogether.

### TIEDs are present in most of the patients

**Figure 3:**
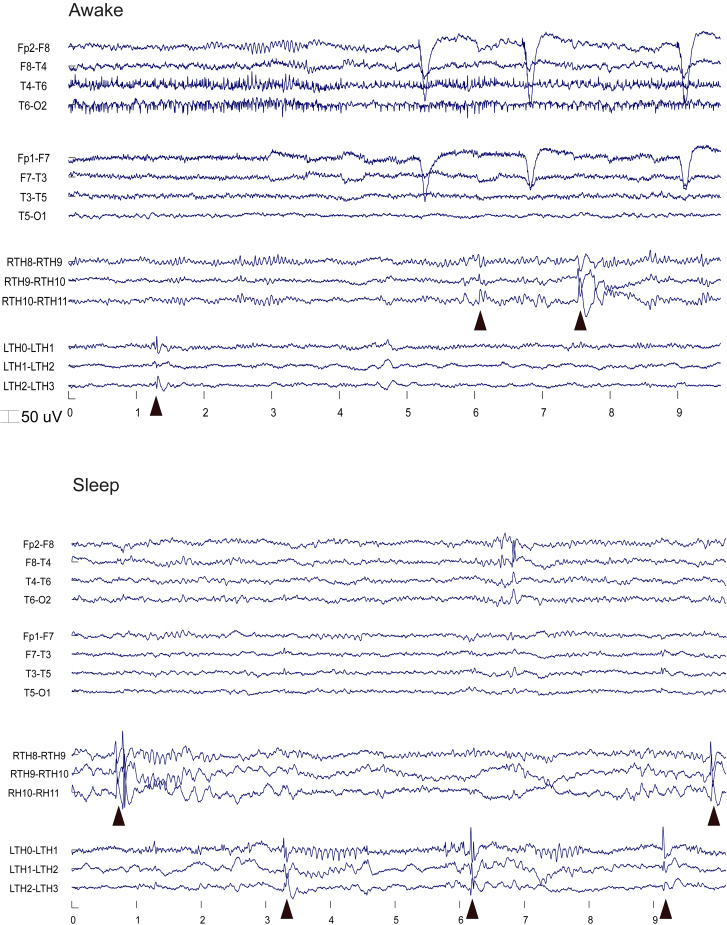
Sleep and awake EEG Triangles show TIEDs.

**Figure 4:**
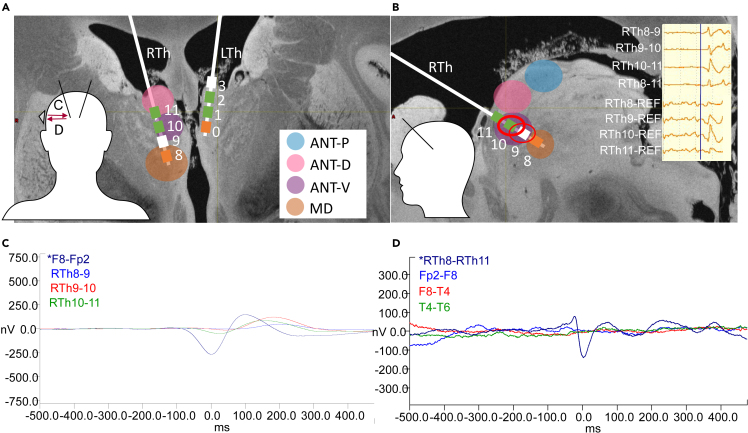
Detection of interictal epileptiform discharges (IED) in the thalamus, and the demonstration of the IED triggered average potential (IED-TP) method (A and B) Subdivisions of the ANT as dorsal (ANT-D: pink), ventral (ANT-V: purple), and posterior (ANT-P: blue) parts and MD (orange) on the schematic representation of the leads in Pt4. (LTh: Left Thalamus; RTh = Right Thalamus). Note that electrode numbering starts from the deepest contacts, with 0 and 8, respectively. Upper right inset on Figure B shows an example of a thalamic IED (TIED) in bipolar and monopolar montages (scale bar: 50 μV). Red circles mark the channels on which IEDs were visually identified (see STAR Methods section for details). Two lower left insets show the schematic head orientations, while inset in A shows a schematic scalp electrode position and averaging directions in C and D. (C) Scalp IED triggered average followed by delayed thalamic potential in Pt9. In this case, the ANT activity of the examined condition was defined as “follower”. (D) Thalamus IED triggered average with no detected IED-TP on scalp recordings in Pt4. Thus, in this case ANT activity was considered to have no connection with the scalp activity.

We detected TIEDs within the thalamus, see Figures 3 and 4B for an example of a typical TIED waveform. Thirteen patients (87%) had TIEDs in the ANT, 7 patients bilaterally, and 6 patients only on one side. The reason for one sided detection was missing contralateral intranuclear lead (n = 5) or real one-sided TIED activity (*n* = 1), in this later case the lead was only a near hit resulting in ANT border—subependymal lead position from a parietal track. TIEDs could be detected in 83% of the intranuclear ANT leads (*n* = 20). Two patients with 3 intranuclear leads had no TIEDs; all of these leads were implanted via the parietal transventricular pathway. All but one patient (*n* = 14) had IEDs on the scalp EEG recordings.

**Figure 5:**
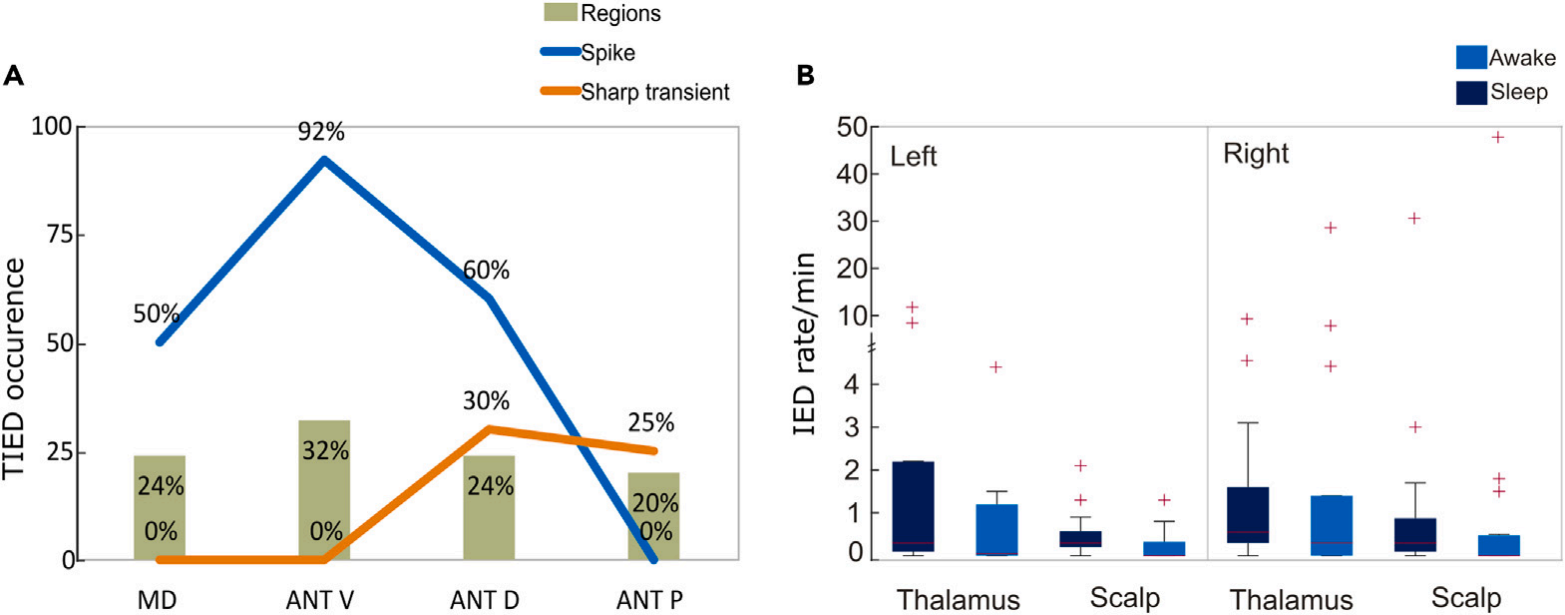
IED occurrence in thalamus and scalp recordings (A) TIED occurrence in percentage within the sampled sub-regions in the thalamus (MD, ANT-V, ANT-D, ANT-P) presenting spikes (blue curve) and sharp transients (orange curve). Green columns show the percentages of the sampled thalamic regions among all the recordings (*n* = 41 regions; sampled with 76 intrathalamic contacts). Table S3. (B) IED frequency in the thalamus and on the scalp separated on the left and right side in sleep and awake state. Boxplots represent median with interquartile range (IQR), whiskers mark the most extreme values within 1.5xIQR. Crosses mark outliers outside this range. Table S2.

Analyzing the circadian distribution of the interictal discharges, scalp IEDs (SIEDs) were recorded in sleep in 93% (*n* = 14), and in awake in 60% (*n* = 9), while TIEDs were detected in sleep in 87% (*n* = 13) and in awake in 73% (*n* = 11) of the patients. The IED rates are shown in Figure 5B. The SIED rate showed the well-known difference between sleep and awake, removing one extreme outlier (Pt 9), we found significantly more IEDs in sleep than in awake (median 0.26/min vs. 0/min; *p* = 0.012, Wilcoxon signed rank test). In the thalamus, similarly more IEDs were observed in sleep without reaching significance (median 0.33/min vs. 0.13/min; *p* = 0.06, Wilcoxon signed rank test). The overall IED rate in the thalamus was not significantly different than on the scalp (median 0.27/min vs. 0.15/min; *p* = 0.08, Wilcoxon signed rank test).

### Localization of the TIEDs within the ANT

We sorted the intrathalamic recordings based on the contact locations. Multiple contacts within the same region in one patient were counted as one. We distinguished the following regions: the mediodorsal nucleus (MD: 24%), and in the further subdivided parts of the ANT: ventral (ANT-V: 32%), dorsal (ANT-D: 24%), and posterior (ANT-P: 20%). The illustration of the used parcellation of the thalamus can be seen on Figures 4A and 4B. We found that the detection of TIEDs showed marked differences between these regions. See Figure 5A for details. Spikes were detected in 50% of the MD, 92% of the ANT-V, and 60% of the ANT-D regions while sharp transients in 30% of ANT-D and 25% of ANT-P recordings.

### Interaction between outcome and TIED detections

**Figure 6:**
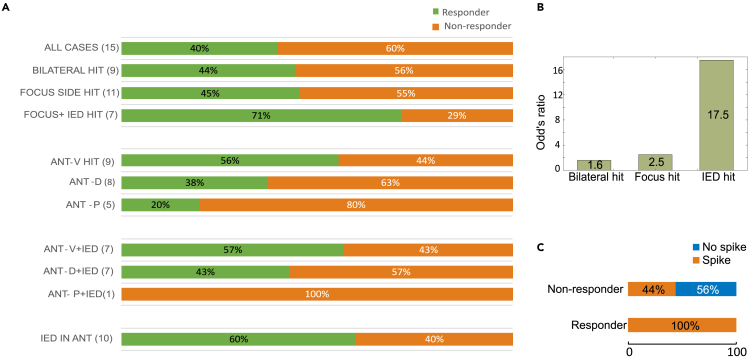
Clinical outcome in function of implantation site and thalamic IED occurrence (A) Percentage of clinical outcome calculated in examined population (All cases), patients with contacts bilaterally within the ANT (Bilateral hit), patients with ANT hit in the dominant side (Focus side hit), patients with hits in different ANT subregions (ANT-V/D/P) combined also with detected thalamic IEDs. (B) Odd’s ratio indicating the association between good clinical outcome (responders) and site of ANT contacts (bilateral hit, focus hit and IED hit). Note that the highest values occur when contacts reside in the relevant side which also presents IEDs. (C) Thalamic IED occurrence in responder and non-responder patients. Table S4.

We compared the response rate (RR) based on different aspects such as lateral dominance, lead position, and detected TIEDs. The overall RR at 1 year follow-up was 40%, while it was 44% in the bilateral hit patients, compared to only 33% of the unilateral hit cases. Based on lateral dominance the RR was 17% in left, 80% in right, and 25% in bilateral cases. We further subselected the patients, combining the lateral dominance with ANT hit. Ideally, in bilateral dominance, we expected bilateral, while in one sided laterality, we expected appropriate side hit. RR was 45% if the lead hit the focus side. Even further, we included TIED detection into this analysis and found that 71% of the patients with TIEDs in the focus side were responders (Figure 6A). We found that odd’s ratio for good outcome was increasingly higher if we hit bilateral ANT (1.6), the focus side (2.5), or TIEDs in the focus side (17.5) (Figure 6B).

Based on TIED detection, we found that all the responder patients had spike-type TIEDs somewhere within the ANT, while only 44% of the non-responders had spike type TIED detected (Figure 6C). The 51 responder patients had significantly more TIEDs than non-responders in sleep and awake (Wilcoxon rank-sum test, *p* = 0.014 and *p* = 0.036, respectively). On the scalp neither sleep nor awake SIED rate was significant (*p* = 0.83 and *p* = 0.76, respectively).

### IED triggered averaged potentials have additional yield

We performed SIED and TIED triggered averaged potential (IED-TP) analysis in eight conditions: back and forth between the scalp and the thalamus, both sides, in awake, and sleep. See Figures 4C and 4D for the illustration of typical IED-TPs. Time lag was assessed between the first peaks of the averaged signals.

In cases of missing IEDs in a particular condition (sleep or awake) or missing ANT hit at one side, the calculation was not possible (see Table S1). Altogether, every patient had at least two conditions with valuable IED-TP data, but we had only one case with all eight conditions available. Based on this, ANT was a preceder in 5%, follower in 55%, and there was no connection in 40%. When the ANT was the follower, the mean time lag was 51 ms (2–180 ms), if the ANT preceded the scalp activity the mean time lag was 18 ms (15–25 ms).

When pooling all conditions, we found that 75% of the examined cases in responder patients fell in the follower category and 18% had no connection, while this comparison was 38% and 59% in the non-responders. This result became more prominent if we analyzed the IED-TPs only in sleep. Using these criteria, the responders were 88%–6% and non-responders 38%–57% followers and no-connections, respectively. The difference between responder and non-responder patients regarding the occurrence of the follower category proved to be significant in both cases (*p* < 0.00001, Fisher exact test).

We compared the time lag in cases of followers in sleep and awake state and found no statistically significant difference between the responders and non-responders (*p* = 0,3516). The average diurnal time lag was 50 ms and 51 ms, respectively. Detailed information of the data can be seen in Table S5.

## Discussion

The participation of the subcortical structures in seizure genesis is a long-standing debate in epileptology.^33^ Epileptic seizures are known to arise from the cortex, but our view on the exact origin of seizure symptoms and electrical phenomena may be hampered enormously by our limited ability to record the activity of subcortical structures.

The thalamus is known to participate in both primary and secondarily generalized seizures.^34^ In focal epilepsy structural alterations can be found in the related thalamic nuclei.^35^ We also know that there are no cortical regions in the brain without strong interconnection to first and higher order thalamic nuclei that form the so-called thalamocortical unit which is considered lately as a basic entity responsible for all the functions that we referred to as cortical processes before.^36^ In this light, it seems to be less significant to distinguish exclusively cortical or thalamic processes.

In our study, we made an attempt to systematically record from the ANT using the therapeutic lead as a diagnostic tool. The results strengthened that different thalamic nuclei such as ANT and MD participate in the epileptic processes. This participation was robust; we found that 87% of the patients produced TIEDs in the ANT regardless of the location (temporal or extratemporal) of the original epileptic focus. The involvement of the different subnuclei of the ANT was not equalized, the ANT-V, that was located the closest to the mamillo-thalamic tract junction, showed the most prominent TIED activity, including 92% of ANT-V recordings. However, our division of the ANT was not identical to the cytoarchitectonics division used in the literature, because, due to the narrow structure of the ANT compared to the dimensions of the lead, we were not able to differentiate the medial and lateral aspects of the nucleus, we hypothesize that the more widespread cortical projections of the antero-ventral and antero-medial subnuclei might explain our findings.^12^^,^^13^^,^^37^

IEDs are the most recorded markers of seizure disorders specific to the epileptogenic cortices and those regions that are functionally connected to it.^38^ Based on this, we hypothesized that TIED activity may prove the pathophysiological connection of the thalamus to the seizure focus, and the stimulation of this area will have better suppressing effect on seizures. Analyzing the 1-year therapy outcome in relation with the preceding TIED recording from the ANT, we found that all of the responders had TIEDs, and conversely, 71% of the focus hit leads expressing TIEDs belonged to responders. Among TIED presenting patients the responders had significantly higher TIED rate than non-responders.

To further investigate the temporal relationship between the thalamus and cortical areas, we made IED triggered averages from the ANT and scalp. Regarding connections between thalamic structures and cortex, the thalamus followed the scalp IED activity in 33 out of 60 potential connections (55%) with an average time lag of 51 ms. In 3 patients (5%), the thalamus preceded the cortex by 18 ms. In 24 cases (40%), no connectivity was noted between thalamus and cortex. Importantly, ANT was a follower in 88% of responders during sleep that was significantly higher than in non-responders, indicating that functional connection between the ANT and the seizure focus is important for the neuromodulatory effect (see Table S5).

These results indicate that the thalamic participation within the epileptogenic process is secondary, which is in line with the original assumption that the primary pathology affects the cortical area. But as the thalamocortical connection forms a functional unit, we think that some sort of acceleration or positive modulation might arise from the thalamic involvement in the pathologic function of the network that can lead to the seizure manifestation. The abruption of this activity at the thalamic site via inhibiting the thalamic activity might disrupt this facilitating effect and the cortical focus will not be able to produce the seizures anymore.

We have proven that ANT and MD produce epileptiform activity in close relationship with that observed on scalp recordings and also in relation with therapeutic outcome. In addition, while ANT involvement in TIED activity was widespread, we found marked differences between the subsection in favor of the ventral part. Hence, our results confirm that detection of thalamic IEDs on implanted electrodes, especially during sleep, might predict the effectiveness of stimulation therapy.

Furthermore, TIED activity might also guide the placement of ANT-DBS in drug resistant epileptic patients to achieve better clinical outcome. The valuable information offered by SEEG and ECoG recordings about the activity of cortical foci might be complemented with thalamic recordings already during presurgical evaluation. On one hand, subjects not eligible for surgery would benefit from the available thalamic data, which might contribute to optimal DBS targeting without an additional invasive procedure. On the other hand, by those receiving surgical treatment, it would be highly relevant to compare thalamic recordings obtained before surgery with the outcome of the treatment. Nevertheless, more data derived from post-implantation period is needed to be able to assess the costs and benefits associated with an additional invasive diagnostic procedure.

### Limitations of the study

The limitation of the study is the relatively low number of the cases and also the relative heterogeneity of the etiologies, although the original selection criterion included a bias for bitemporal epilepsies. The responder rate is lower in our population than in other ANT-DBS publications reported in the literature that also limited our findings.^6^^,^^8^ Also, the scalp electrodes provide a vague insight to the activity of the cortex, especially considering the mesial temporal structures, so the actual relationship of the seizure focus to the thalamus cannot be studied in detail with this approach.

## STAR★Methods

### Key resources table

**Table:** 

REAGENT or RESOURCE	SOURCE	IDENTIFIER
**Software and algorithms**		

BRAIN QUICK® EEG Software	Micromed S.p.A., Mogliano-Veneto, Italy	https://micromedgroup.com/
FMRIB Software Library v6.0	Woolrich et al.^40^	https://fsl.fmrib.ox.ac.uk/
Mathlab 2019	The MathWorks, Inc.	www.mathworks.com
Neuroscan SCAN 4.5	Compumedics, Victoria, Australia	Compumedics Neuroscan – World Leader in Functional Neuro-imaging
EEGlab	Delorme et al.^41^	https://eeglab.org/
Excel	Microsoft Corp.	www.microsoft.com
Inkscape	Open Source Scalable Vector Graphics Editor	www.inkscape.org

**Other**		

DBS system	Medtronic Corp.	www.medtronic.com

### Resource availability

#### Lead contact

Further information and requests for code and data should be directed to and will be fulfilled by the lead contact Dániel Fabó (fabo.daniel@gmail.com).

#### Materials availability

This study did not generate any materials.

#### Data and code availability

• The dataset of surface and intracranial anterior thalamus EEG used in this publication is not made publicly available due to privacy related agreements but can be requested from the lead contact.
• This paper does not report original code.
• Any additional information required to reanalyze the data reported in this paper is available from the lead contact upon request.

### Experimental model and study participant details

#### Participants

We implanted fifteen therapy resistant epilepsy patients with bilateral ANT-DBS lead system (Lead model: 3389, Medtronic, MN, USA). The subjects (6 male) ranged in age from 23 to 64 years (median 39). The onset of epilepsy was between 1 to 34 years, and the duration of epilepsy varied from 7 to 38 years (median 23 years). All patients belong to the white race.

#### Ethics

The patients were provided with written informed consent according to the ICH – GCP and the declaration of Helsinki (Intézeti Kutatásetikai Bizottság, IKEB 6/2016).

### Method details

#### Patient selection

The patient selection was based exclusively on clinical grounds and included patients without the possibility of resective epilepsy surgery. During the selection process bitemporal cases had preference over others based on previous reports. All patients underwent preoperative epilepsy evaluation. Ten patients had been examined with invasive electrodes preoperatively, in the others scalp video-EEG was sufficient for localisation of the seizure focus. Resective surgery was rejected in all the cases. The decision of the lead positions and routes was based entirely on neurosurgical grounds.

Therapy outcome was assessed one year after the start of the DBS stimulation, average seizure count was compared between periods of 3 months before implantation and 3 months prior the 1-year postoperative follow-up, all the seizure types were counted together. Patient by patient detailed average seizure reduction (SR) rate is visible in the Table S1. Patients with more than 50% seizure reduction were defined as responders. Response rate (RR) was measured as the responders ratio per the total number of patients. According to the protocol the antiseizure medication therapy was kept unchanged for this 1-year post-DBS period, except the cases where worsening of the seizure status was measured (Table S3). All the responder patients were classified as Engel III, while non-responders were categorized as Engel IV.

#### Planning, imaging and monitoring

Preoperative planning of proper trajectories had been carried out using a Medtronic S7 Planning Station and Cranial software on high resolution contrast T1, axial STIR, coronal STIR, and 3D gradient echo sequence with two inversion recovery times by visualizing the mammillothalamic tract as anatomical guidance. Thirty leads with four contacts each (120 contacts altogether) were implanted (15 patients, 2 per patient). The electrodes were implanted in different trajectories: 18 leads from frontal transventricular, 10 from parietal transventricular and 2 from lateral approach.

After lead implantation an extension cable (Medtronic type 3389-40) was attached to the lead and externalized. The patients were transferred to the epilepsy monitoring unit (EMU) video-EEG department and a modified junction cable (37086-60) was attached in order to connect the leads to the EEG, just like a stereo-EEG electrode. Additional scalp EEG electrodes were placed on the head according to a modified 10-20 system. Those electrodes interfering with bandages (usually some out of F3, F4, C3, C4) were left out. EEG and thalamic LFPs were sampled together using extracranial G2 reference.

The patients spent two consecutive nights in the EMU. No medication was tapered. Spontaneous LFP were recorded with BrainQuick System Evolution Video-EEG system (Micromed S.p.A., Mogliano-Veneto, Italy). LFP and EEG were sampled at 2048 Hz. The video-EEG session was conducted according to the clinical standards examining epilepsy patients.

Postoperative electrode locations were identified based on preoperative MRI (3D T1, 1x1x1 mm isotropic voxels) and postoperative CT (1 mm slice thickness) co-registration (FMRIB Software Library v6.0 Linear Registration Tool, FSL FLIRT, FMRIB, Oxford, UK). CT images were acquired at least 6 weeks after surgery for electrode localization to exclude pneumocephalus related artifacts. Contacts were identified using three-dimensional Euclidean vector calculations, during which the most distal, a proximal part, and the exact dimensions, contact size and distance of the electrode were taken into consideration. See Figure 2 for contact localizations. Parts of the ANT were divided visually as ventral (ANT-V), dorsal (ANT-D) and posterior (ANT-P). These parts were not identical to the antero-medial, antero-ventral, and antero-dorsal subnuclei of the ANT, rather reflect a parcellation of the nucleus only in the sagittal plane (see Figure 4B) for demonstration.

Active contacts were selected according to proper placement within the ANT to achieve the most feasible therapeutic effect and were modified according to the side effect profile. The contacts located in the ANT were subsequently stimulated; the stimulation parameters were 2-5 V, 130-140 Hz, 60-90 μs pulse width, using 1 min ON, 5 min OFF duty cycle. According to the protocol the selected contacts were not modified further until one year follow up, but in cases where patient seizure status worsening or side effects were detected, changes in settings were necessary, see Table S3.

#### Analysing EEG

EEG and LFP analysis were performed both in awake and sleep state. Twenty minutes of artifact free slow-wave sleep from the second night and also 20 minutes of awake recordings were selected for IED analysis. Segments were selected to be apart from seizures for at least 2 hours. We found that 20 minutes were a consequently reproducible amount of EEG in every patient that fulfilled all above mentioned criteria. We omitted REM periods due to the low number of TIEDs. TIEDs were reviewed off-line and detected visually by expert epileptologists (ZsJ, DF). Among TIEDs we identified two sub-categories. Spike was defined as consisting of a fast (shorter than 50 ms) component with a prominent amplitude from the background viewed both in bipolar and monopolar montage followed by a slower wave component. The contact with maximum amplitude of the fast component was identified in monopolar montage. In case of an outstanding graphoelement in bipolar montage that lacked either phase reversal in bipolar montage, or outstanding amplitude in monopolar montage, regardless of the duration of its fast component was registered but coined sharp transient (ST). Figure 4B illustrates a representative example for an identified IED on all thalamic contacts, using monopolar and bipolar montages, respectively. Scalp IEDs (SIED) were visually identified on bipolar scalp montage.

SIED and TIED triggered averages were calculated to measure peak to peak IED amplitudes and time lags between scalp and thalamic recording sites. The analysis was performed using Neuroscan SCAN 4.5 software (Compumedics, Victoria, Australia). Detectable peaks around ±500 ms window around averaged IEDs were labeled as IED-Triggered average Potentials (IED-TP). In case of the presence of IED-TPs, we calculated the time lag between maximum peaks of the averaged IED and IED-TP waveforms. For averaging, the IED peaks were set to 0ms, and time-lag values were measured as absolute ms values of the IED-TP peak. ANT was coined as “follower” relative to scalp recordings in two cases: if after the SIED averaging process the thalamic IED-TP had positive latency (followed it), or if after the TIED averaging process, the scalp IED-TP had negative latency (scalp preceded the thalamus). In the converse relationships the ANT was considered a “preceder”. Conditions, where no detectable IED-TP peaks were found, were labeled as “no connection” (Table S5). The IED-TPs and time lags were calculated back and forth between the thalamus and scalp, in sleep and awake states, on each side, so every patient had 8 possible conditions to analyze. Conditions with missing IEDs were omitted.

### Quantification and statistical analysis

As IED frequency did not follow normal distribution, non-parametric statistical tests were applied. For comparison of paired data (sleep vs awake and thalamus vs scalp data of the same individuals) Wilcoxon signed rank test, while for unpaired data (responder vs non-responder patients), Wilcoxon rank sum test was used. Responder and non-responder patients were also compared in terms of the temporal relationship between thalamus and scalp recordings. Time lags were compared by independent t-test. Occurrences of a certain type of relationship were compared using Fisher exact test. pP-value <0.05 was considered to be statistically significant. Data analysis was carried out using MatLab and Microsoft Excel software.
